# Clinical Microscopy for the General Practitioner

**Published:** 1895-08

**Authors:** Hugh H. Young

**Affiliations:** San Antonio, Assistant Surgeon in the Johns-Hopkins Surgical Dispensary. Pathologist to the Thomas Wilson Sanatarium for the Children of Baltimore


### TEXAS MEDICAL JOURNAL.

ESTABLISHED JULY, 1885.

PUBLISHED MONTHLY.—SUBSCRIPTION $2.00 A YEAR.

VOL. XI. AUSTIN, AUGUST, 1895. NO. 2.

### Original Contributions.

For the Texas Medical Journal.

### CLINICAL MICROSCOPY FOR THE GENERAL PRACTITIONER.

BY HUGH H. YOUNG, A. M., M. D., SAN ANTONIO,

Assistant Surgeon in the Johns-Hopkins Surgical Dispensary. Pathologist to the Thomas Wilson Sanatarium for the Children of Baltimore.

ON LOOKING over the leading medical journals one is struck with the amount of space which is now given to bacteriology, but alas! how little of it can be put into practical use by the busy practitioner.

Many books on bacteriology are at hand, but amid the multitude of things which can only be attempted in a well equipped laboratory, it is hard for the practicing physician to find anything that is of practical value to him, and he often gives up all attempt.

Recognizing this, the writer will endeavor to point out the simpler methods of clinical microscopy, which can be made use of with the outlay of a small sum, and a little time. These are largely the methods employed at the Johns-Hopkins Hospital, and can generally be found in books on the subject, but some of them have not been published, and many are much simplified.

The first requisite, of course, is a microscope. This should have three lenses, a low and a high for urinary work, etc., and an oil-immersion lens for bacteriological and blood examinations. It is not necessary to send to Europe for this, as a splendid microscope with low power (⅔) and high power (⅙) and an oil-immersion lens ($\frac{1}{12}$) can be gotten for about $110. This instrument will answer every requirement for general work. With the outlay of $3.00 for an Arnold's steam sterilizer (which every surgeon ought to have), and $16 for an oven or incubator, almost any bacteriological work could be done. With these at hand the ordinary media are not difficult to prepare, but they can be easily procured (ready prepared in tubes) at small cost, from supply houses and laboratories, if desired.

But the object of this article is to show how, with only a microscope at hand a good many valuable bacteriological diagnoses can be made.

*Standard Solutions.*—Of the vast number of these used only three are necessary for general use, and are as follows:

Loeffler's alkaline methylene blue:

℞ Concentrated alcoholic sol. methylene blue..ʒiiiss.

Caustic potash in 1-10,000 sol. in water......ʒiss.

The solution of methylene blue is made by filling a small bottle about one-fourth full of methylene blue powder, filling with alcohol, corking tightly and shaking well. After standing twenty-four hours it is again shaken well, and then twenty-four hours later when a sediment has fallen to the bottom and the liquid above is a clear blue, it is filtered and added to the solution of caustic potash, and kept in two ounce bottles. These are more convenient if they have small glass syringes fitting in their necks in place of glass stoppers.

This stain is of particular value for the bacillus diphtheriæ. For other bacteria the carbolic fuchsin stain, which is more durable, can be substituted. The methylene blue stain should be prepared fresh every month.

*Ziehl's Carbolic Fuchsin Solution.*—

℞ Distilled water.................ʒii.

Carbolic acid..................gr. l.

Alcohol.... ................ʒi⅔

Fuchsin (in substance)........gr. x.

This stain is more durable than methylene blue, and can be used for all bacteria, and in the first of the two stains used for tubercle bacilli. The second stain is

*Gabbett's Acid Methylene:*—

25 per cent. sulphuric acid sol. in water...ʒii.
Methylene blue (substance).......grs. x. xx.

It is only used in staining for tubercle bacilli.

The platinum "needle," which is generally used to inoculate tubes, and to prepare mounts of bacteria, and pus for microscopical examination can be made by simply heating one end of a small glass rod in a flame, and inserting a small platinum wire into the softened end. The end of the wire (which should be two inches long), is more serviceable if bent into a circle the size of a pin head.

The simplest way to prepare a mount is to sterilize the platinum "needle" (or loop) in a flame, and having inserted it into the pus or pus cavity, to make a thin smear on the surface of a clean glass slide. As soon as this is dry, the slide is carried rapidly across the flame three times, to fix the smear. It is now stained by dropping on a sufficient amount of methylene blue or carbolic fuchsin solution, which is washed off at the end of four to twenty seconds, according to the activity of the stain. It is not necessary, and much simpler not to put any cover glass on the slide, but to apply a drop of immersion oil, after the slide has been dried between blotters, and examine with the power.

I have gone very much into detail, but it seemed necessary for those who have not had any laboratory experience.

Those bacteria whose recognition is of clinical importance are principally the pyogenic cocci, the genococcus, the bacillus diphtheriæ and bacillus tuberculosis.

*The pyogenic cocci* are principally the streptococcus pyogenes, the staphylococcus pyogenes aurens, and less often the albins.

It is often important to know early, both for prognosis and treatment, whether an acute inflammatory condition is due to the streptococcus or the staphylococcus—the one liable to lead to extensive cellulitis, lymphangitis and grave systemic infection, the other perhaps only a localized abscess. In abscesses of a subacute character where it is hard to say whether the process is tubercular or not, an examination of the pus is often of great assistance. If tubercular, a mount made from pus drawn off with a sterile syringe, may occasionally show tubercle bacilli, but generally no bacteria at all. But if due to the staphylococcus, this will be found here and there, often single, in pairs, and bunches of three, four, five, etc., but rarely in the large typical "grape bunch" arrangement seen from cultures.

The streptococcus too does not show the characteristic morphology in pus, being rarely seen in chains of more than seven or eight elements, and most frequently in twos.

The presence of *actiosomyces* and *anthrax* can also be made out microscopically when not suspected, as in a recent case at the Hopkins dispensary, in which I found the characteristic chains of anthrax bacilli in the serous fluid from an obscure œdema around the right eye.

Loeffler's methylene blue and the carbolic fuchsins are equally suitable for staining the pyogenic cocci, the nuclei of the pus cells taking the same stains as the bacteria, while the bodies of the cells are unstained. These can be stained by a saturated solution of eosin in water, if desired.

*Diphtheria.*—The ease with which this organism can be diagnosed and the importance of early injections of antitoxine make it almost the duty of the practitioner to examine all suspicious cases microscopically.

The sterilized platinum (or a twist of cotton on an applicator) is rubbed on the apparent membrane, and withdrawn so as not to touch anything else if possible. A good smear is then made on a slide as above, and stained with Loeffler's methylene blue. It is best to put on a large quantity of the stain, heat to the boiling point over a flame and then washing off well, to blot dry, put on a drop of oil and examine.

The diphtheria bacilli will appear as straight or slightly curved rods, with rounded or pointed ends; but more frequently irregular wedge, club, and spindle shapes appear. In preparations stained with Loeffler's methylene blue many of these irregular rods have circumscribed spots in their substance which stains more deeply than others, thus showing a somewhat coarse transverse striatum, or large, deeply staining granules in a lightly stained bacillus.

If organisms, corresponding with the above description, are found in very great number, you may be tolerably certain of diphtheria. But several organisms, which habituate the mouth, resemble it so strongly at times that most authorities advise a culture to prove the diagnosis. For this purpose, culture tubes of Lœffler's blood serum mixture are the best, and can be procured, at about five cents a tube, from the larger laboratories and supply houses. They are inoculated by rubbing the platinum loop over the surface of the media. If these are now put in an incubator for twenty-four hours, the diphtheria bacilli will grow out as small grayish white colonies with irregular margins, which are generally larger than growths of the other bacteria. A mount made by spreading one of these colonies with the loop on a slide, will show the characteristic irregular rods described above. If a very small drop of water be previously placed upon the slide, the spreading will be greatly facilitated.

If no incubator is at hand, the tubes should be placed in a warm place, as on the mantle next to a warm chimney. If no blood serum tubes are obtainable, a *hard boiled egg* may be utilized. The large end—which has the bubble—is cracked and the shell removed for a space the size of a cent. With sterized tissue forceps the membrane is torn and drawn to one side and the flat end of the egg inoculated by rubbing with the loop. The egg in then placed in a clean tumbler, cracked end down, and set in a warm place on the mantle. Colonies, similar to those on blood serum, will grow out on the surface of the inoculated egg, and if they show the typical morphology microscopically, a positive diagnosis of diphtheria can be made.

This simple expedient can be used with very good results, and many cases diagnosed within twenty-four hours, and long before any constitutional symptoms have developed.

The *gonococci* can be readily stained in pus smeared on a slide, with methylene blue or carbolic fuchsia. It is shaped like a biscuit (seen on its side) and occurs in pairs—the flat surfaces of the buscuit shapes being nearest each other and parallel. Another characteristic is, that these biscuit-shaped pairs are found in great numbers in the pus corpuscles—lying around the nuclei in the protoplasm, which is very faint, unless counter-stained with eosin.

The shape and location of the gonococci (within the pus cells) as given above, can be considered sufficiently diagnostic. The other more difficult methods of diagnosis are not necessary for ordinary cases.

The detection of the gonococci is often especially valuable in medico-legal cases, in cases of urethritis, vaginitis, etc., in children, and also in cases of cystitis and pyonephrosis. In a case here, lately, of general infection, following gonorrhœal pyonephrosis, the organism was found in the blood and also in the vegetation of an acute endocarditis, and cultures made on human blood serum.

The *tubercle bacillus* is so well known to every one that it would seem superfluous to say anything about it; but the staining methods generally in vogue are so complicated that it may not be amiss to describe Gabbett's method, which is much simpler.

In collecting sputum, it is best not to save sputum just after meals, as particles of food may be taken for the memmular masses which contain the bacteria in greatest number. These masses are best seen when the sputum is spread out on a glass plate, when they appear as grayish-white opaque particles. These are picked out with a sterilized bonnet pin and smeared on the surface of a slide in as thin a film as possible. In this way several particles can be smeared on the same slide in different places, which is then allowed to dry, when it is passed through flame three times.

A good quantity of the Zuhl-carbolic fuchsia solution is then poured on the slide, covering well the smeared surfaces. Holding the slide in forceps, it is held over the flame until brought to the boiling point, when the stain is quickly washed off, allowing the water to run until it comes away clear. Then dry the slide between blotters and pour on an abundance of Gabbett's acid methylene blue (see above). After thirty seconds this should be washed off. The slide should now have a blue color, but if it still shows spots of red, methylene blue must be again applied, until these disappear entirely. This method is by far the simplest. Tubercle bacilli, if present, will show as long, curved bacilli, stained *red*, while every other organism and cell should be *blue*.

In tuberculosis of the genito-urinary tract, the bacilli can often be found in the urine, but the constant presence of a bacillus in the smegma, which has the same morphology and staining properties at the tubercle bacillus, render it necessary to obtain the urine from the bladder by means of a sterilized catheter. A small amount of the urinary sediment is spread thinly on a slide and allowed to dry. After passing through flame, it is stained in the same manner as sputum.

The *microscopical examination of urine* for casts, etc., is too well known to require mention. The quickest and most convenient way, is to spread the sediment thinly over a large part of the slide, and examine with the low power (⅔) without the use of a cover glass. In this way casts can be detected very quickly, and a final diagnosis as to their variety made by using the high power (⅙).

*Blood.*—Microscopical study of blood has of late assumed such an important role that a few remarks about it may not be *mal apropos.*

Blood to be examined is best taken from the lobe of the ear, which is first wiped clean and then pierced by a quick stab with a needle—preferably a straight Hagadom—with a cutting edge. The first drop of blood having been wiped off, a second is squeezed out and quickly touched with a clean cover-glass, which is held in readiness in the other hand. The cover-glass is immediately placed on a slide, and if the manipulations have been rapid enough the blood will spread out into a thin film. If not, the corpuscles will be bunched, in rouleaux, and crenated.

The piercing of the ear is painless, and it is well to practice on some kind friend, until a rapid technique is acquired.

The plasmodia of malaria can be readily seen in blood prepared in this way. The organism of simple tertian fever will be found generally in a red blood corpuscle which is more or less decolorized—thus being in marked contrast with the other corpuscles which are yellowish green in color. The organism appears as transparent refractive, often irregular mass, generally surrounded by small pigment granules in active dancing motion, and occupying a smaller or larger portion of the decolorized corpuscle, according to the age of the parasite—its cycle of life being forty-eight hours. After rupture of the corpuscle the young plasmodia can be seen free—extra-cellular—while others have entered fresh corpuscles, to begin again their destructive cycle of life.

The microscopical diagnosis of malaria is so easy that it ought to shed light on many or the doubtful fevers of the South. Every one interested will read with pleasure the exhaustive monograph by Tayer in the Johns-Hopkins hospital reports.

The above method is also of great value in showing the poilslocytosis in pernicious anæmias, and the large increase of leucocytes in leukæmia, and furnishes the diagnosis.

The *amœba coli* plays such an important part in the ætiology of many of the obscure chronic dysenteries, especially in the southern latitudes, that its detection is of great importance—especially so, since a few injections of quinine are so destructive to the organisms as to cure a chronic case in a few days.

The amœba is found more easily in necrotic flakes which are passed with the discharges. These appear as grayish-white flakes or shreds, and are picked out with a bonnet pin, placed on a slide and teased apart. If too dry, a drop of water at about the temperature of the body, may be added, and then a cover-glass put on, and pressure applied until the specimen spreads out into a thin layer. Close the diaphragm of the microscope so as not to have too much light, and under the one-sixth power the amœba appears as a large transparent, refractive mass, several times as large as a leucocyte, and showing the slow, worm-like amœboid movements, if the slide be kept warm. This is best done by removing the slide every minute or so and warming gently over an alcohol flame, but not allowing it to become hot to the finger.

This brief outline is a bare introduction to the possibilities of clinical microscopy, but if he has simplified it so as to induce one to attempt this important and greatly neglected branch of medicine, the writer will feel justified in having written it.

---

For the Texas Medical Journal.

